# A Comprehensive Overview on COVID-19: Future Perspectives

**DOI:** 10.3389/fcimb.2021.744903

**Published:** 2021-09-14

**Authors:** Rashmi Rana, Ankit Tripathi, Naveen Kumar, Nirmal Kumar Ganguly

**Affiliations:** Department of Research, Sir Ganga Ram Hospital, New Delhi, India

**Keywords:** COVID-19, clinical manifestations, epidemiology, future prospects, pathogenesis, treatments

## Abstract

The outbreak of COVID-19 has proven to be an unprecedented disaster for the whole world. The virus has inflicted billion of lives across the globe in all aspects—physically, psychologically, as well as socially. Compared to the previous strains of β-CoV genera- MERS and SARS, SARS-CoV-2 has significantly higher transmissibility and worst post-recovery implications. A frequent mutation in the initial SARS-CoV-2 strain has been a major cause of mortalities (approx. 3 million deaths) and uncontrolled virulence (approx. 1 billion positive cases). As far as clinical manifestations are concerned, this particular virus has exhibited deleterious impacts on systems other than the respiratory system (primary target organ), such as the brain, hematological system, liver, kidneys, endocrine system, etc. with no promising curatives to date. Lack of emergency treatments and shortage of life-saving drugs has promoted the repurposing of existing therapeutics along with the emergence of vaccines with the combined efforts of scientists and industrial experts in this short span. This review summarizes every detail on COVID-19 and emphasizes undermining the future approaches to minimize its prevalence to the remaining lives.

## Introduction

The influence of viruses and viral infections on human history has been broadly described by the **social history of viruses** ever since the modifications in human behavior during the Neolithic period around 12,000 years ago (Baranowski et al., 2001; Fuchs et al., 2019). This was the period when humans began to expand their agricultural communities and an exponential increase in the spread of viruses leading to becoming endemic was observed the most. With the rapid globalization and anthropogenic activities with time, pathogenic transmission has escalated across the globe and resulted in viral pandemics (Fuchs et al., 2019). It was the mid-19th century that was remarkably known for pathogenic viral outbreaks and their multiplex associations with humans and animal species. This further leads to cross-species transmission, posing a high threat to human health and well-being  (Ye et al., 2020).

Later at the beginning of the 21st century, it was observed that due to the rapid globalization and human activities, pathogenic transmission across continents has escalated and resulted in several pandemics, especially viral pandemics (Ye et al., 2020). The pandemic caused by old diseases, namely plague, cholera, and yellow fever in addition to some emerging diseases such as Ebola, Zika, severe acute respiratory syndrome (SARS), middle east respiratory syndrome (MERS), and COVID-19, makes it the most ferocious century in human history (Ong et al., 2020). As per the reports, these viral pandemics have caused significant mortalities and majorly affected the international economy over the last three decades (Ong et al., 2020). For example, the Ebola viral disease (EVD) identified in 1976 in Central Africa for the first time and its outbreak in 2014–2016 has resulted in more than 40% mortality in West Africa (Control and Prevention, 2014). SARS-CoV infection was first identified in 2003 and has known to be originated from bats and transmitted to humans *via* palm civets (host) in Guangdong Province, China; there were 8422 reported cases including the mortality rate of 11% in 26 countries (of the International, 2020). Similarly, MERS-CoV also originated in bats, transmitted through dromedary camels as an intermediate host reported in 2494 cases with 858 deaths (mortality rate 34%) in 27 countries  (Omrani et al., 2015).

Now the biggest threat that the world is facing today is the outbreak of novel coronavirus (COVID-19) that originated in Wuhan, Hubei Province, China, in December 2019 and rapidly spread over the rest of the world in a short time (Boni et al., 2020). It can best be characterized by pneumonia-like symptoms that may further extend up to major hypoxia and several cardiovascular complications (Boni et al., 2020). Coronaviruses are enveloped, positive-sense, single-stranded RNA viruses belonging to the Coronaviridae family and a leading cause of acute respiratory, hepatic, and neurological diseases with variable severities in vertebrates (Wang et al., 2020). They are referred to be the common human pathogens with the tendencies of fleeting recombination and mutation (Wang et al., 2020). It is due to the presence of crown-like spikes on the periphery of these viruses, popularly called coronaviruses (Boni et al., 2020; Wang et al., 2020). These coronaviruses are segregated into four distinct genera based on phylogenetic clustering, namely alpha coronavirus (αCoV), beta coronavirus (βCoV), gamma coronavirus (γCoV), and delta coronavirus (δCoV) (Huang et al., 2020). Among these, α and βCoVs (mainly found in bats and rodents) are known to infect humans; however, γ and δCoVs (found in birds) are known to infect mainly aves and mammals including pigs (Paim et al., 2019). In addition to affecting a vast majority of humans by crossing the inter-species barrier, βCoVs (i.e., SARS-CoV and MERS-CoV) have been marked with the highest mortality rates among all the classes of coronaviruses (Huang et al., 2020). Structurally they are composed of four major proteins: (a) the spike (S) protein, (b) the nucleocapsid (N) protein, (c) the membrane (M) protein, and (d) the envelope (E) protein, playing a pivotal role in mediating the attachment of the virus to the host receptor, its subsequent fusion, and accelerating virus assembly within the host system (Samidurai and Das, 2020). Until 2003, barely two human CoV (HCoV) strains, HcoV-OC43 and HcoV-229E, were recognized, but from 2003 to 2021, the world has experienced havoc and an exponential increase in mortality rates due to the emergence of 5 other deadly strains of coronaviruses: HcoV-NL63, HcoV-HKU1, severe acute respiratory syndrome corononavirus (SARS-CoV), middle east respiratory syndrome coronavirus (MERS-CoV), and SARS-CoV-2, which may cause fatal respiratory infections in humans (Ivanov and Ziebuhr, 2004; Ye et al., 2020). Due to the rapid spread of the disease and its manifestations (i.e., enhanced mortality rate) caused by the newest corona strain viz. SARS-CoV-2 originated from Wuhan, China, in December 2019 has raised the concerns of researchers and clinicians concerns across the world (Long et al., 2020). As a result, the World Health Organization (WHO) in February 2020 has named it coronavirus disease 2019, abbreviated as COVID-19, and on March 11, 2020, the situation was declared a pandemic (Long et al., 2020).

The statistical data on COVID-19 has reported around 4,995,996 confirmed cases of SARS-CoV-2 infection along with 327,821 deaths in 216 countries, and the number is increasing exponentially daily (Nižetić, 2020; Sharma et al., 2020). China, which is an epicenter of SARS-COV-2, has reported 84,520 confirmed cases with 4645 deaths, the United States of America (USA) has 1,528,186 confirmed cases including 92,000 deaths, and India has a maximum number of confirmed cases at 22,362,920 and 242,000 deaths (Sharma et al., 2020). To control COVID-19’s superspread event and its impact on global health care infrastructure, the WHO has substantiated on early diagnosis, prevention, social distancing, proper sanitization, and complete lockdown-like strategies. Additionally, the guidelines approved by various national and international authorities about the dos and don’ts have been made available to national and international platforms (Saadat et al., 2020). Apart from this, rigorous efforts being made by our researchers and medical practitioners to disseminate accurate details mimetic to etiology, pathogenesis, clinical course, and protective measures for the widespread COVID-19 disease across the communities, one of the fastest and reliable sources is real-time counts infected cases worldwide (Nižetić, 2020).

In corroboration with the recent editorial in *Lancet* prioritizing the spread of reliable, adequate, and independently scrutinized data and information related to COVID-19 disease among the general audience, the present review abridges all the scientific findings related to the COVID-19 outbreak in one place and thereby minimizes the effort of readers in going through the enormous studies available online.

## Emergence and Evolution of SARS-CoV-2

Since the outbreak of COVID-19 disease in December 2019 in Wuhan, China, the interest of epidemiologists has piqued in assessing the rationale behind the eruption of SARS-CoV-2 in humans, including the involvement of animal reservoir, endemic circulation, co-infection, recombination events within RNA segments, and its time of divergence from animal species (Decaro et al., 2021).

In December 2019, when the cases of pneumonia were epidemiologically related to the open-air seafood market in Wuhan, China, the local authorities in China provisioned an epidemiological alert and issued a complete lockdown for a couple of weeks (Decaro et al., 2021). After rigorous research and clinical implications, in January 2020, the scientists at Wuhan obtained a complete genome sequence from the infected people and obtained around 80% sequence similarity with SARS-CoV, confirming pneumonia to be a SARS-induced condition (Zhou et al., 2020). Initially, this novel human pathogen was placed in the Sarbecovirus subgenus of the Coronaviridae family, the family in which SARS falls (Boni et al., 2020). The virus is responsible for more than 8,200 cases from 2002–2003. Later, by mid-January 2020, the virus did super-spread within China, and by mid-March 2020 was labeled as pandemic status (Cucinotta and Vanelli, 2020). This, in turn, enhanced the concern of the medical fraternity to prevent its spread and, at the same time, the researchers across the world were busy identifying the strain affecting millions of lives (Cucinotta and Vanelli, 2020). With the subsequent studies and ongoing reports the virus was named SARS-CoV-2 by the International Committee on Taxonomy of Viruses (ICTV) study group and also named hCoV-19 by Wu et al. (Wu et al., 2020a).

For the first time in a consortium on virological.org led by Zhang on January 10, 2020 (GMT), the advent of the first genome sequence of SARS-CoV-2, Wuhan-Hu-1 assisted the researchers to understand the ancestry of this novel coronavirus (SARS-CoV-2) (Boni et al., 2020; Wu et al., 2020b). Later the data from the bioinformatics analysis has evidenced the homology between SARS-CoV-2 and other members of the coronavirus family, especially with the betacoronavirus 2B (Boni et al., 2020). Thus further studies have been undertaken on SARS-CoV-2 by considering it to be a new member of betacoronavirus 2B lineage infecting humans (Boni et al., 2020). Upon aligning the full-length genome sequence of SARS-CoV-2 and obtainable genomes of beta coronaviruses, scientists observed around 96% of sequence identity within the genomes of SARS-CoV-2 and SARS-like BatCov and RaTG13 coronaviruses. This indicates the bat origin of SARS-CoV-2 or, in other words, SARS-CoV-2 has been naturally evolved from bats (Boni et al., 2020; Naqvi et al., 2020; Xiao et al., 2020; Zhou et al., 2020).

Concurrently, studies have also suggested Malayan pangolins (*Manis javanica*) to be the possible host in the emergence of SARS-CoV-2 infection in humans (Xiao et al., 2020). Pangolins, the scaly ant-eaters belonging to the mammalian order Pholidota, are among the illegally trafficked mammalian species used for food and medicine purposes (Lam et al., 2020). Due to the extensive manhandling of these pangolins, researchers have decided to conduct a study on frozen tissue samples (blood, lungs, and intestine) obtained from 18 Malayan pangolins during an anti-smuggling task by Guangxi Customs officers (Lam et al., 2020). In particular, data from high through-put RNA sequencing has confirmed the Malayan pangolins to be the intermediate host of coronaviruses to humans, and later the readouts from sequence similarity search have demonstrated nearly 85.5–93% identity in the sequences of pangolin coronavirus genome and SARS-CoV-2 (Lam et al., 2020). Hence, the local authorities in China have decided to remove pangolins from wet markets to avoid further zoonotic transmissions (Boni et al., 2020; Lam et al., 2020).

## Portal of SARS-CoV-2 Entry in the Host Cell

The respiratory tract is considered to be the prominent portal for the ingression of viruses into the mammalian system, due to its direct contact with the external environment (Matrosovich et al., 2004). Therefore, the principal symptoms and complications of SARS-CoV-2 are observed in the respiratory tract at its primary stage (Belser et al., 2013). The viral particles encapsulated in the droplets or aerosols are released from a COVID positive individual when inhaled by a healthy and uninfected person, and the SARS-CoV-2 adheres to the specific cell-surface receptor for the viral protein. In due course it enters into the endosomes and, finally, the viral and lysosomal membranes fusion occurs (Peiris et al., 2003; Belser et al., 2013).

The process of SARS-CoV and SARS-CoV-2 coronaviruses entrance into the host is facilitated by the host cells Transmembrane protease serine 2 (TMPRSS2) and lysosomal proteases (especially cathepsins) through two independent mechanisms: proteolytic cleavage of ACE2 receptor which stimulates viral uptake and cleavage of coronavirus spike glycoproteins which turns on the glycoprotein for host cell entry (Zhang et al., 2021). The host cell entry mechanism of these coronaviruses has been extensively studied and found to be almost similar in the case of both SARS-CoV and SARS-CoV-2 (Zou et al., 2020). The virus entry into the cell is mediated by the spike proteins anchored onto the virus surfaces. The spike protein on a mature virus consists of three receptor-binding S1 heads existing on top of trimeric membrane fusion S2 stalk. Once the virus is inhaled by the healthy individual and enters into the respiratory tract, it is the S1 subunit of spike protein with receptor-binding domain (RBD) which first recognizes the human angiotensin-converting enzyme-2 (hACE-2) as its receptor (Zheng, 2020; Zou et al., 2020). In general, hACE-2 is a membrane-bound protein expressing in several human cells, namely respiratory tract (abundant in the lower respiratory tract), vascular endothelium, cardiovascular tissue, renal tissue, and intestinal epithelia (Wang et al., 2020). After the recognition of hACE-2 by S1, the proteolytic activation of SARS spike protein at S1/S2 boundary is triggered by the activity of cell surface protease TMPRSS2 and lysosomal proteases (cathepsins) (Wang et al., 2020). Their activity causes the dissociation of S1 from S2, and the segregated S2 molecule further undergoes dramatic conformational changes. This in turn activates the glycoprotein for host cell entry, causing ingression or release of viral RNA into the host cytoplasm, followed by a translation of new viral proteins and affecting nearby cells in the vicinity (Walls et al., 2020; Wang et al., 2020).

The cellular entry mechanism for SARS-CoV and SARS-CoV-2 are reported to be almost similar, with a difference in receptor (hACE-2) recognition and binding potential of RBD units in S1 glycoproteins on the surface of SARS-CoV and SARS-CoV-2. The binding affinity of SARS-CoV-2 with hACE-2 is known to be comparatively higher than that of SARS-CoV. Apart from this, the presence of an extra proprotein convertase (PPC) motif in the spike protein of SARS-CoV-2 also distinguishes it from SARS-CoV (Berry et al., 2004; Wang et al., 2020).

## Epidemiological Traits of SARS-CoV-2

As per the literature, bats and pangolins are reported to be the primary and intermediate reservoirs for the SARS-CoV-2 strain of coronavirus infecting humans (Zheng, 2020). Apart from this, the animals residing in proximity with humans, especially cats, ferrets, and even golden hamsters, are at high risk of SARS-CoV-2 transmissions (Sharun et al., 2021). Initially, it was reported that the expected roots of transmission for SARS-CoV-2 are mainly droplets and fomites exchange between a nCoV infected and an uninfected, healthy individual (Jayaweera et al., 2020). Later a study has also stipulated the emergence of virus from the surrounding environment (air-borne), potentially affecting an uninfected person upon inhaling the aerosols emitted by an infected person while exhaling, sneezing, shouting, coughing, etc. (Gralton et al., 2011; Liu et al., 2017). The authors have also quoted that SARS-CoV-2 could remain stable in the aerosols for 3 h (van Doremalen et al., 2020). This report on the airborne transmission of respiratory viruses has been considered as the dominant mode of spread, as it was even difficult to demonstrate on ground levels compared to those of droplets and ferrets mediated transmission (van Doremalen et al., 2020).

As per the recent reports, this new human pathogen (SARS-CoV-2) can continue to stabilize in the digestive tract for a longer duration than in the respiratory tract (Xu et al., 2020). Studies on humans have noted the presence of viral RNAs in the excreta of infected people for more than 33 days after they have been detected as COVID negative (Arslan et al., 2020; Wu et al., 2020d). This particular finding substantiates another unprecedented mode of viral transmission, i.e., fecal-oral route of viral distribution in the environment (Wu et al., 2020d). The viral transmission through the fecal-oral route was further confirmed when children with COVID-19 positive tests have reported negative results in nasopharyngeal swabs while their rectal swabs indicated a consistently positive result for COVID-19 infection (Yeo et al., 2020; Wu et al., 2020d). Noteworthy, the discharge of COVID-19 patients’ fecal matter may increase the potential risk for wastewater treatment plants (WWTPs), as the virus could embed into the fecal matter and settle in WWTPs (Arslan et al., 2020). Studies have indicated the presence of SARS-CoV-2 in sewage samples in seven cities of Netherlands and Schipol airport at Amsterdam between February and March 2020 (Arslan et al., 2020).

Initially, it was known that SARS-CoV-2 cannot be distributed through intra-uterine vertical transmission from a pregnant woman to an infant, but later the studies have changed the notion by demonstrating the possibility of vertical transmission of the virus in a newborn with few neurological disorganizations whose mother tested positive with SARS-CoV-2 during the last trimester of her pregnancy (Chen et al., 2020; Wang et al., 2020). Likewise, a case was reported wherein anti-SARS-CoV-2 IgM antibodies and IL-6 levels were noted to be comparatively higher than the normal neonates indicating the likelihood of transplacental transmigration of virus from COVID-19 positive mother to the neonate (Dong et al., 2020). There is an availability of a relatively good amount of data stating the perinatal transmission of SARS-CoV-2 in pregnant women, but the chances are quite low as compared to SARS-CoV-1 and MERS (Fan et al., 2020; Parazzini et al., 2020; Wang et al., 2020).

**Figure 1:**
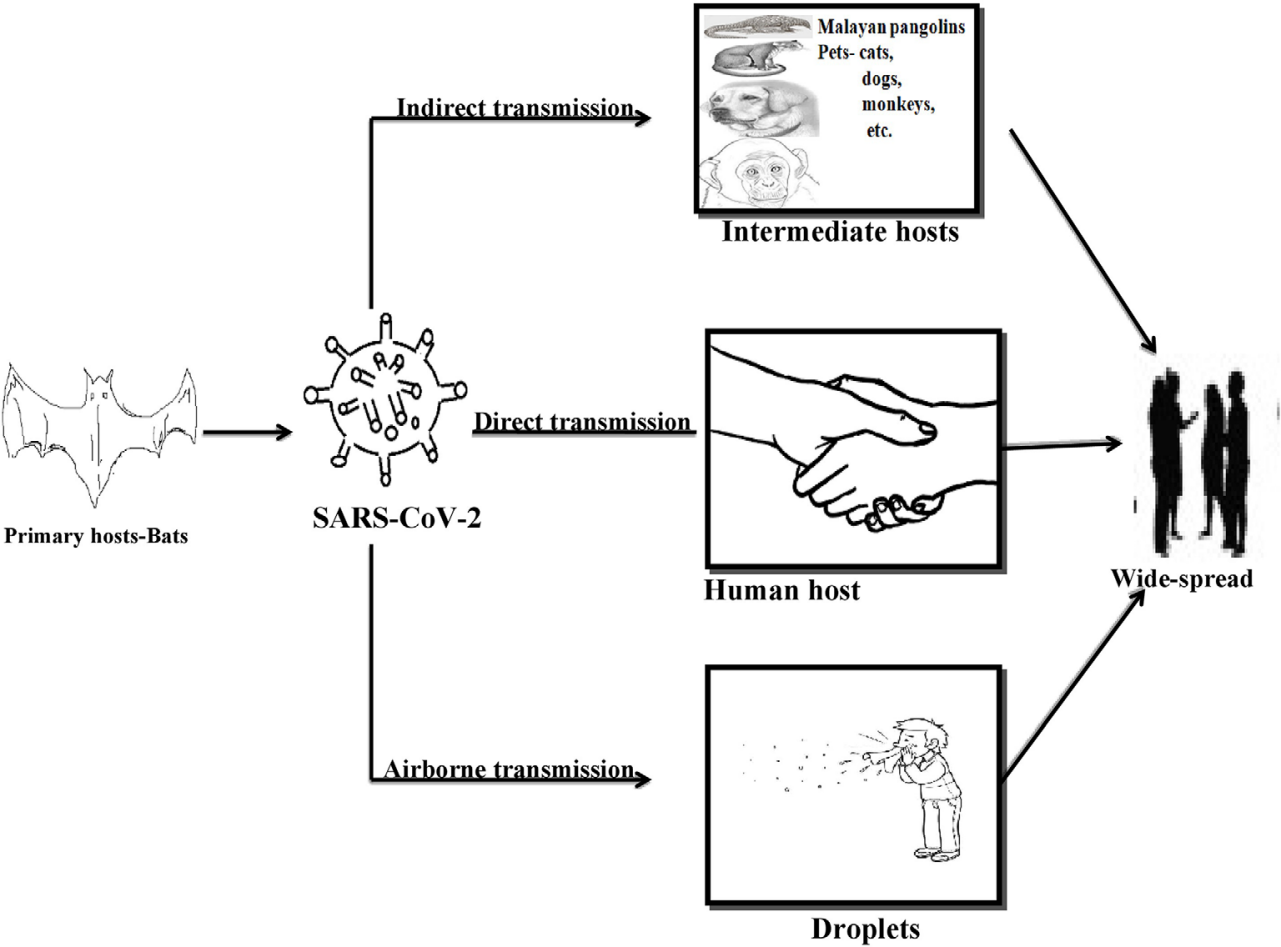
The potential hosts and possible routes of SARS-CoV-2 dissemination.

Among all the common and major spread routes for the SARS-CoV-2 virus is direct contact, i.e., person to person contact, public gatherings, and/or crowding at one place as compared to the fecal-oral transmissions, vertical transmissions, and aerosol-mediated spread of virus among the community (Ghinai et al., 2020b; Ghinai et al., 2020a). This viral transmittance is not only limited to human-to-human transmissions but also the pattern of animal to human, human to animal, and animal to animal transmissions being frequently observed in recent days. The best-known instances for animal to human transmission of the primary and intermediate reservoir of SARS-CoV-2 for humans is bats and Malayan pangolins as discussed above. However, transmissions from humans to animals was confirmed when a study indicated the resemblance in viral genetic sequences of SARS-CoV-2 diagnosed in two dogs with that of human SARS-CoV-2 virus (Sit et al., 2020). Hence it was discovered later that animals like tigers, cats, and dogs are on the higher edge of getting infected with the SARS-CoV-2 virus when residing in contact with an infected person for longer (Singla et al., 2020). As the virus can be transmitted from one infected person to another, a similar trend has been observed in the case of animals when for the first time a SARS-CoV-2 positive cat has affected the naïve cat with the same virus and an adult SARS-CoV-2 positive ferret affected a naïve ferret *via* close contact (Wang et al., 2020). See **Figure 1**.

## Variants of Coronavirus

There is a general tendency of viruses including COVID-19 causing SARS-CoV-2 to evolve and gradually change over time (Abdool Karim and de Oliveira, 2021). During the replication process, these viruses every-so-often undergo changes in the genetic code termed as “mutation,” giving rise to a new strain of virus called “variant” (Abdool Karim and de Oliveira, 2021). Mutation in viruses is neither new nor unprecedented, it is a commonly occurring phenomenon in almost all viruses having RNA as a genetic material (Manrubia and Lázaro, 2006). It is mostly the geographic separation events that may result in genetically different variants (Manrubia and Lázaro, 2006).

The data from high throughput sequencing analysis has confirmed around 20 mutation events in the genome of SARS-CoV-2 collected in October 2020 over the first strain sequenced in January 2020 (Wuhan-Hu-1) (Fang et al., 2021). The virus has been reported evolving at a rate of ∼1.1 × 10^−3^ substitutions per site per year, corresponding to one substitution every 11 days approximately (Martin et al., 2021). This contrasts with the mutations in the HIV occurring at a rate of ∼4 × 10^−3^ substitutions per site per year (Andrews and Rowland-Jones, 2017). Based on the aforementioned details, the US government interagency established a variant classification scheme that classifies SARS-CoV-2 variants into three distinct groups:

**(A). Variants of Interest (VOI):** It can best be defined as an isolate of SARS-CoV-2 with genotypic and/or phenotypic changes compared to the reference genome. It is a variant with discrete genetic markers associated with inducing alterations in receptor binding, minimized neutralization by antibodies generated against previous exposure of viruses, affecting diagnostics and treatment strategies (Covid, 2021). The threshold for defining a VOI is quite low, to support surveillance efforts.

To date, eight different VOIs for nCoV-2 have been reported in the literature. These variants are an outcome of a common mutation, i.e., D614G, first documented in the United States of America (USA) during the initial phase of the pandemic (Control and Prevention, 2021). The variant with D614G mutation in SARS-CoV-2 spike glycoprotein curtails S1 shedding and enhances viral infectivity compared to the viruses without this mutation or with different mutations (Control and Prevention, 2021). Following is the list of VOIs known till date:

**(i). B.1.526:** This variant of SARS-CoV-2 was first identified in November 2020 and reported to spread at an alarming rate in New York. DNA sequencing analysis has confirmed the presence of B.1.526 sequence in approximately 27% of the total populatiobn of New York City (Annavajhala et al., 2021). The variant arose due to **E484K** and **S477N** mutations in the receptor-binding domain upraising complications associated with resistance to vaccine-elicited and therapeutics (Annavajhala et al., 2021).

**(ii). B.1.526.1:** The variant for the first time was identified in New York City (October 2020). It is a sub lineage of B1.526.1 with **T95I** and **D253G** spike mutation in nCoV-2 original strain (Wang et al., 2021). Like the parent strain (B.1.526), this variant also imparts potential resistance against monoclonal antibodies and reduction in neutralization by post-vaccination sera (Wang et al., 2021).

**(iii). B.1.525:** This variant was identified in December 2020 for the first time in Nigeria mainly and the sequence analysis studies later have confirmed its appearance in UK and France as well. It is also called the Nigerian strain of SARS-CoV-2. The variant is thought to be an outcome of E484K with H69–V70 deletion and Q677H mutation in the S1 domain of viral spike protein. The B1.525 mutant strains are attributed to increased transmissibility, virulence, and immune escape (Ozer et al., 2021).

**(iv). P.2:** The whole-genome sequencing studies have reported their occurrences in Brazil mainly and in some regions of Manaus since April 2020. This variant is a sublineage of **B.1.128** lineage with **E484K** point mutation in the receptor-binding domain of SARS-CoV-2 S1 glycoprotein. Presence of E484K mutation in the virus-induced reduced neutralization by polyclonal antibodies in convalescent sera (Naveca et al., 2021b; Nonaka et al., 2021; Resende et al., 2021).

**(v). B.1.617:** It is the most prominent mutation in India now, which was detected for the first time in Maharashtra, India, in February 2021. It is often called a double mutant of novel coronavirus due to two prominent mutations: **E484Q** and **L452R** (Challen et al., 2021). The presence of this variant has triggered the transmittance and drug or vaccine resistance capacity of SARS-CoV-2 in infected people. Later on, UK detected three different but genetically resembling variants of COVID-19 that emerged in Indian: **B.1.617.1, B1.617.2,** and **B.1.617.3** found to be adversely affecting the U.K., U.S., and Israel health sector (Challen et al., 2021; Ferreira et al., 2021).

**(B). Variants of concern (VOC) or emerging variants:** As per the document issued by WHO on February 25, 2021, outlining the description of VOCs and VOIs, VOC can be expounded as a VOI with a noticeable increase in spread, virulence, and demonstrable impacts on diagnosis/treatment/vaccines (Harper et al., 2021). The mounting data on the initial variant of concerns have identified some of them, and the research is still underway to identify the presence of other unknown VOCs:

**(i). B.1.1.7:** It was first identified as VOC in December 2020 by COVID-19 Genomics (COG)-U.K. consortium, i.e., COG-UK. B.1.1.7 was recognized as the most frequently spreading variant across the UK during the nationwide lockdown; however, other strains usually demonstrate a significant reduction in their transmission by lockdown or social distancing (Frampton et al., 2021). Thus with a rigorous evaluation of retroactive data, the researcher has confirmed the existence of the variant in circulation since September 2020. The variant is also known as 20I/501Y.V1. Studies on B.1.1.7 stipulate that it is one of the well-versed and highly sequenced VOCs with the highest transmissibility (at a rate of between 40% to 70%), infectivity (30% to 50% higher than other strains), and demonstrable mortality rates (61% to 67%) due to mutation in the Y501 region of S1 protein of the virus (Galloway et al., 2021).

**(ii). P.1:** This particular variant has been detected in Japan by their surveillance system in 4 travelers who had returned from Brazil (Naveca et al., 2021a). The variant was noted to be emerged due to N501Y mutation associated with higher binding affinity to hACE receptor, **E484** mutation linked to drug/monoclonal antibodies/vaccine resistance, and **K417N/T** mutation responsible for imparting higher receptor binding affinity to virus in combination with N501Y mutation in the spike protein of the virus (Naveca et al., 2021a).

**(iii). B.1.1351:** The variant is popularly known as **“**20H/501Y.V2” or “**South African** variant.” It was first identified in Nelson Mandela Bay, South Africa, in October 2020, after frontline clinicians were notified about the increased frequency of cases to the Network for Genomic Surveillance in South Africa (NGS-SA), which in turn promoted genomic investigations and analysis (Ellis, 2021). The variant is known to have multiple mutations in spike protein, especially the **K417N, E484K**, and **N501Y** mutations. These mutations make it a variant of concern as it exhibits enhanced transmissibility and resistance to vaccines (Ellis, 2021).

**(iv). Cluster 5:** Danish public health authorities first identified this variant on mink farms in Denmark and Netherlands on November 5, 2020 (Larsen and Paludan, 2020). After this, Denmark decided to halt all farmed mink in Denmark (Larsen and Paludan, 2020). The emergence of the Cluster 5 variant is due to the notable Y453 F mutation in the SARS-CoV-2 S1 domain, imparting resistance against neutralizing antibodies (Larsen and Paludan, 2020).

**(C). Variants of high consequences:** The presence of such variants ensures a considerable reduction in the effectiveness of preventive measures or medical countermeasures (MCMs) as compared to the variants in circulation. The relieving part is that currently there is no trace of the presence of such threatening variants reported or that has come forward from any region of the world (Control and Prevention, 2021).

It is very important to keep an eye on the circulation of these variants and to work efficiently on their preventive measures and vaccine suppression strategies. Moreover, it is equally important to keep track of any further mutation in the nCoV-2 genome by genomic surveillances and sequencing methodologies.

## Clinical Manifestations

Patients with COVID-19 may have an extensive range of clinical manifestations. The clinical attributes of COVID-19 may vary from patient to patient ranging from asymptomatic to acute respiratory distress syndrome (ARDS) (Nepal et al., 2020). In general, the disease manifestations of nCoV-2 are dominated by a respiratory condition known as interstitial pneumonia (Nepal et al., 2020). A person infected with SARS-CoV-2 will initially experience fever, sore throat, dry cough, headache, fatigue, restlessness, myalgia, anosmia, and dysgeusia (Nepal et al., 2020). Later it may progress to mild to moderate pneumonia followed by hypoxia, and if left undiagnosed and untreated, then it may further lead to severe complications such as acute respiratory disease syndrome (ARDS) and systemic inflammatory response syndrome (SIRS), and multiorgan failure (MOF) and/or shock (Nepal et al., 2020).

Regardless of respiratory symptoms, unrestricted SARS-CoV-2 infection may stimulate a severe immune reaction called a “Cytokine storm,” in which a body dissipates too many cytokines in the blood very quickly and uncontrollably (Zhai et al., 2020). As a result, the production of neutrophils, proinflammatory cytokines (IL-1β, IL-6, TNF-α, etc.), and chemokines (Ccl1, CXCl10, Ccl3, etc.) exceeds the levels of anti-inflammatory cytokines in the body, which in turn leads to multiorgan damages (Fu et al., 2021). The majority of patients together with the asymptomatic ones are reported to exhibit diffused bilateral pneumonia surrounded with ground-glassy opacity either progressing or coexisting with consolidation (Cui et al., 2020). Histological examinations have also evidenced that the lower respiratory tract holds a higher overall viral load than the upper respiratory tract (Wölfel et al., 2020). Besides this, pathological findings in the infected lungs have also clearly indicated the appearance of proteinaceous exudates in lung tissues as well as in BALF, development of pulmonary edema, bilateral diffuse alveolar damage (DAD), interstitial thickening, infiltration of T cells or inflammatory monocytes, etc. compared to a healthy lung (Wölfel et al., 2020). Moreover, COVID-19 patients have also been marked with relatively low levels of lymphocytic T cells (CD4^+^ and CD8^+^) and natural killer (NK) cells, i.e., overall low levels of lymphocyte counts in the blood profile (Varchetta et al., 2021). Two of the most prominent reasons for low lymphocytes are hypokalemia (low potassium levels) and hypophosphatemia (low sodium levels), induced due to the impact of SARS-CoV-2 on patients ACE-Angiotensin-II (ACE-Ang-II) that prevents the degradation of intact Ang-II within the system. As a result, aldosterone production triggers promoting to frequent vomiting, diarrhea, and urination. This in turn affects lymphocyte production in the infected person (Kordzadeh-Kermani et al., 2020). In addition to this, people with co-morbidities like diabetes, hypertension, hypothyroidism, chronic lung diseases (COPD, ALI, etc.), any malignancies, even obesity are at high risk of severe COVID-19 infection (Chen et al., 2020). In conformation with retrospective studies during the first wave of COVID-19 (i.e., unmutated strain), aged people (>50 years) were known to be at high risk, but the variant of SARS-CoV-2 as a leading cause for the second wave has affected youngsters mainly as compared to children and the aged once (Ioannidis et al., 2021). Hence aging cannot be precisely considered a factor for COVID-19 infection (Ioannidis et al., 2021).

Besides the involvement of the respiratory tract, the involvement of other vital organs has also been reported in the literature during infection either directly or indirectly (Catapano et al., 2021). According to the literature emergence of interstitial pneumonia in COVID-19 patients is an additive effect of respiratory complications and GI tract symptoms (Su et al., 2020). The presence of dense hACE-2 receptors on the epithelial cells of the GI tract promotes the viral ingression into the GI tract and causes GI associated abnormalities, namely vomiting, diarrhea, nausea, abdominal pain, etc. (Su et al., 2020). Data from the recent retrospective studies on COVID-19 has indicated that around 10% of the mortalities occurred because of low cardiac reserves, especially coronary artery disease and heart failure (Guzik et al., 2020). The laboratory findings suggest the spike in hs-Troponin-I levels and significant abnormalities in electrocardiogram (ECG) are the leading factors of nCoV-2 associated cardiac injuries, namely acute coronary syndrome (ACS), myocarditis, arrhythmias, venous thromboembolic episodes, and pericarditis (Guzik et al., 2020). Several case reports and series of surveys on hospitalized COVID-19 patients so far evidenced the acute kidney injury (AKI) to be a pivotal reason for COVID-19 related deaths (Lee et al., 2021). Similar to the lungs, kidneys are also the potential site of action for SARS-CoV-2 viruses due to the presence of enriched ACE-2 receptors (Lee et al., 2021). The clarity came after pathological examinations which demonstrated the dramatically higher counts of D-dimer, hematuria, proteinuria, serum creatinine, and microalbumin in the blood and urine samples of SARS-CoV-2 affected patients (Kordzadeh-Kermani et al., 2020). However, the effect of nCoV-2 infection on mammalian hematologic mechanism came into existence after autopsy reports obtained from six COVID-19 patients unveiled the SARS-CoV-2- mediated deterioration of spleen and lymph nodes, implicating the abnormal hematopoiesis, coagulopathy, and clear sign of thrombocytopenia to be the major cause of death (Connors and Levy, 2020; Fox et al., 2020; Qu et al., 2020).

According to Cai and Huang et al. (April 2020), more than 40% of COVID-19 patients have exhibited abnormal liver functioning and liver injuries due to increased levels of alanine aminotransferase (ALT) and aspartate aminotransferase (AST) (Cai et al., 2020). Recently there have been some insights pointing towards the higher affinity of SARS-CoV-2 towards hACE-2 receptors present on cholangiocytes, leading to cholangiocytes deregulation followed by the induction of systemic inflammatory response, a major factor responsible for liver injury in the majority of cases (Kordzadeh-Kermani et al., 2020). Besides this elevated alkaline phosphatases, higher Gamma-glutamyltransferase (γ-GT) and lactate dehydrogenase (LDH) levels in the hospitalized COVID-19 patients have confirmed the impact of COVID-19 on the patient’s liver (Cai et al., 2020; Kordzadeh-Kermani et al., 2020). The list of organs and vital parameters getting affected by COVID-19 hasn’t been terminated here. The breakthrough occurred when the findings from Hamburg, Germany, confirmed the deleterious effects of SARS-CoV-2 on the mammalian endocrine system, by putting forward a report stating around 68% of the severe COVID-19 cases have presented critically low levels of testosterone and dihydrotestosterone along with elevated levels of estradiol in the males mimetic to higher IL-6 counts. Females with COVID-19 disease demonstrated higher testosterone levels correlated with IL-6 increase (Schroeder et al., 2020). Researchers from the retrospective cohort have already declared the direct impact of nCoV-2 on Leydig cells, a leading cause of testosterone secretions during stress or infection (Zou et al., 2020).

Later, as the knowledge on COVID-19 and its clinical features continued to expand, the observational studies came forward with shreds of evidence on neurological symptoms in the patients infected with COVID-19 (Kordzadeh-Kermani et al., 2020). The clinical implications of COVID-19 on the neurological system have been evidenced for the first time in a clinical report from Wuhan, China, when a 62-year-old severe COVID-19 patient admitted to a local hospital was shown to develop intracerebral hemorrhage which later progressed to intracranial hemorrhage, and the patient died finally (Li et al., 2020b). In corroboration to this was a clinical investigation of a 79-year-old male without any medical background of hypertension and a 54-year-old woman with a medical history of hypertension suffering from COVID-19 and admitted to a local hospital in Iran, presented with fever, dry cough, and acute loss of consciousness in initial stages of infection. With the progression in severity of infection, the CT brain examinations unveiled an immense intracerebral hemorrhage in the right hemisphere along with intraventricular and subarachnoid hemorrhage in the former case and bilateral sub-acute basal ganglia hemorrhage in the latter case (Nepal et al., 2020). Subsequently, many other studies and case reports have confirmed the invasion of SARS-CoV-2 through the hACE-2 receptors on nasal and oral cavities leading to impaired functioning of sensory neurons, exhibiting neuromuscular symptoms, confirming the involvement of all three nervous systems (CNS, ANS, and PNS) in COVID-19 mediated mortalities (Mao et al., 2020). The nCoV-2 induced complications in CNS are confirmed by the commonly observed symptoms like headache, dizziness, ataxia, epilepsy, and impaired consciousness in COVID-19 patients (Mao et al., 2020). Intense nerve pain, skeletal muscle injury, cranial polyneuritis, neurosensory hearing loss, dysautonomia, neuro-ophthalmological disorders, Guillain-Barré syndrome, and similar signs represent PNS manifestation in COVID-19 infected patients (Andalib et al., 2021). However, the effect of COVID-19 on ANS is linked to cytokine storm as a response to viral ingression within the system. This exceedingly higher level of proinflammatory cytokines due to activation of the sympathetic system leads to vagal stimulation in order to produce anti-inflammatory cytokines to counter the higher levels of proinflammatory cytokines. As a result, symptoms like orthostasis hypotension, postural orthostatic tachycardia condition, and vasovagal syncope have been observed, which confirms the abnormal ANS (Dani et al., 2021).

**Figure 2:**
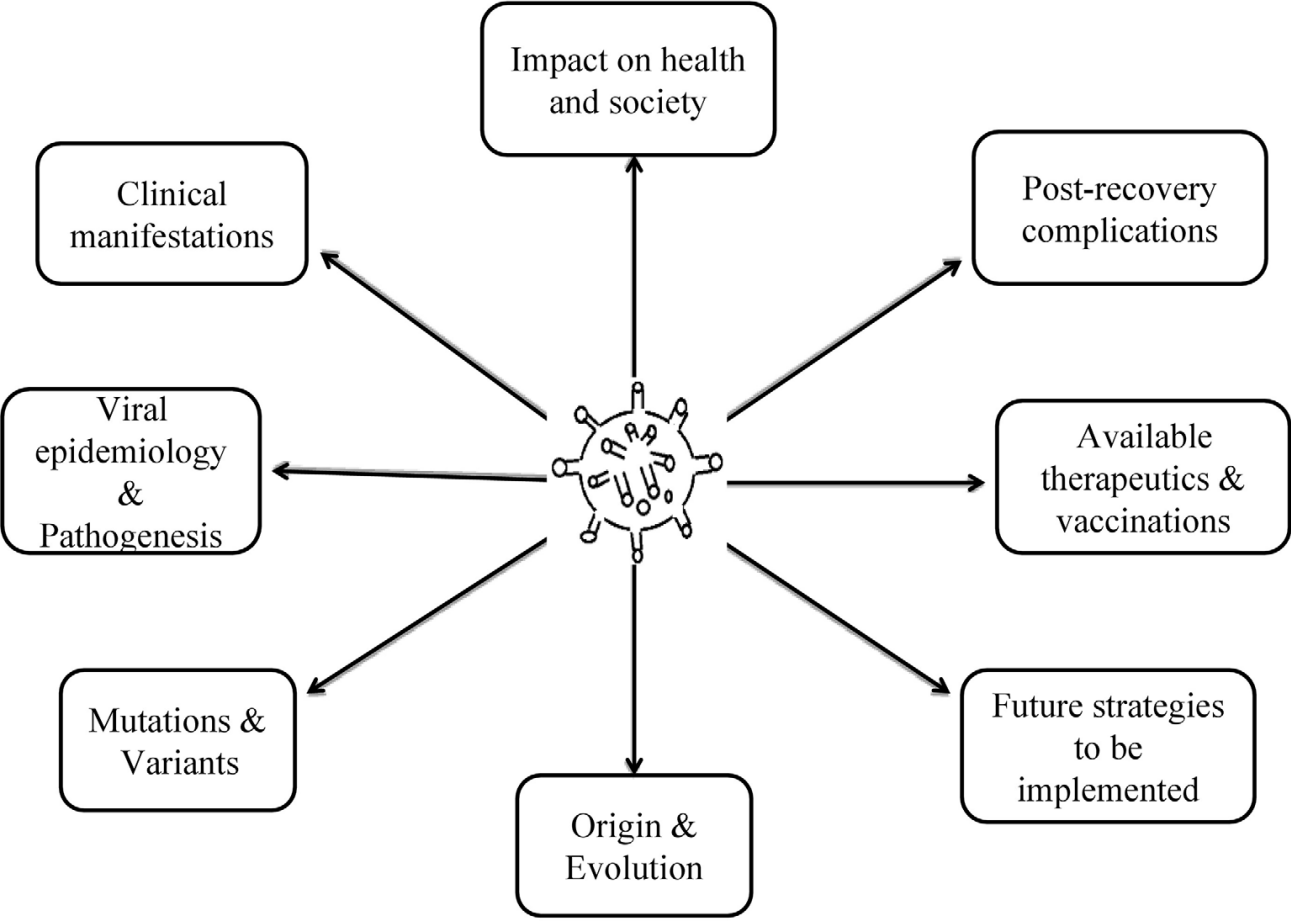
Systematic overview of the complete article.

Under mild COVID-19 conditions, the home-quarantined patients have also experienced erythema, papules, rashes, abnormal scaling patterns, the appearance of chicken-pox like vesicles followed by itching, and burning symptoms on the dermal tissues infrequently (Tsankov and Darlenski, 2020). Of note, references have also indicated the ocular manifestations in the patients with SARS-CoV-2 characterized with lacrimal infection, epiphora, chemosis, and conjunctival hyperemia (Recalcati, 2020). COVID-19 patients have shown interestingly higher levels of LDH, leukocytosis, CRP, and prolactin, which have been considered to be pivotal factors for ocular manifestations of COVID-19 (Wu et al., 2020c) (**Figure 2**).

## Post-COVID-19 Complications

The majority of COVID-19 patients recover within a week or two after infection; on the other hand, some of them have been noticed to experience moderate to severe post-COVID conditions (Silva Andrade et al., 2021). The data from COVID-19 hospitals from different nations suggest multiple health issues extending from a week to a month, even in people who did not have symptoms during COVID-19 infection (Silva Andrade et al., 2021). Predominantly it is the fatigue, muscle ache, headache, chest pain, cough, reduced performance, anxiety, lack of concentration, and depression-like symptoms being experienced in the patients with the “Long COVID-19” condition (Silva Andrade et al., 2021).

After diving deep into the health status of patients diagnosed with negative COVID-19, symptoms such as acute disseminated encephalomyelitis, Guillain-barre syndrome (GBS), acute necrotizing hemorrhagic encephalopathy (ANHE), acute neuropathy, etc. arising due to deregulated immune response, cranial involvement, and impaired central and peripheral nervous system are seen nowadays as a post-COVID condition and have become a matter of concern Montalvan et al., 2020; (Sedaghat and Karimi, 2020; Shahmirzaei and Moghadasi, 2021). Apart from these multiorgan effects, especially autoimmune conditions and multisystem inflammatory syndrome (MIS), i.e., a clinical condition in which edema occurs in different regions of the body due to elevated proinflammatory cytokines production have also been reported by the patients after a week of recovering from COVID-19 (Ramos-Casals et al., 2021). Further progress in scientific and clinical investigations, pieces of evidence related to the post-COVID multiorgan condition with an extensive spectrum of manifestations of the disease, is brought into the knowledge. Examples of these multiorgan systems include rapid hair loss, feces with viral load for longer times, persistent palpitation, dyspnea, bone demineralization, uncontrolled diabetes, restrictive pulmonary physiology, elevated D-dimer, and COVID-19-associated nephropathy (COVAN) condition (a foremost pattern of renal injury in the majority of the African population) (Mokhtari et al., 2020; Velez et al., 2020).

Our local health sectors and doctors are required to provide maximum attention to the COVID-19 patients who were on ventilators and hospitalized for longer durations as they are the ones who have consistently been reported to present the most complicated manifestations of the disease such as severe weakness, post-intensive care syndrome (PICS), post-traumatic stress disorder (PTSD), and the most deadly “mucormycosis” infection (Garg et al., 2021; Smith and Rahman, 2020).

**Mucormycosis** is one of the devastating but rare fungal infections caused by exposure to a group of mucor moulds named mucormycetes. These mucormycetes, members of Mucorales order, are the cluster of fungi existing throughout the environment predominantly in soil rich in decaying organic matter such as animal drugs, composite piles, dead leaves, etc. (Lehrer et al., 1980). These fungi are more common in soil than in the air; similarly, they are noted to be more active in summers than winters or springs (Richardson and Rautemaa-Richardson, 2020). Among these mucormycetes, it is the *Rhizopus* and *Mucor* species that are commonly known to cause mucormycosis (Richardson and Rautemaa-Richardson, 2020). As per the literature, the majority of us are encountered with microscopic fungal spores on a regular basis, as it is almost impossible to 100% circumvent the contact with mucormycetes. Although it is not really harmful to most people, for those with a weak immune system and recovering from some critical pathologies and still breathing in mucormycetes spores, then the possibility of infection in the sinuses, brain, lungs, and to other body parts may occur (Spellberg et al., 2005). This particular fungal infection is noted to be life-threatening in severely immunocompromised individuals or patients with diabetes mellitus (Spellberg et al., 2005).

Depending on its clinical features and anatomical localization, mucormycosis is broadly classified into six distinct classes: (1) Rhinocerebral or rhino-orbitocerebral mucormycosis, (2) pulmonary, (3) cutaneous, (4) gastrointestinal, (5) disseminated, and (6) uncommon presentations (Spellberg et al., 2005). People infected with mucormycosis exhibit the presence of substantial angioinvasion followed by blood vessel thrombosis and tissue necrosis (Ibrahim et al., 2012). As a result, penetration through endothelial cells lining blood vessels and their deterioration is observed commonly (Ibrahim et al., 2012). Based on a retrospective cohort study on the mucormycosis, involvement of some predisposition conditions has been noted and reported in the present review; rhinocerebral, pulmonary, and disseminated muccormycosis are commonly known to affect those with uncontrolled diabetes mellitus (specifically in those with ketoacidosis), extensive burns, iron overload, solid malignancies, treatment with glucocorticosteroids, or patients with neutropenia (Skiada et al., 2020). GI mucormycosis may arise due to malnutrition (Skiada et al., 2020). However, cutaneous/subcutaneous mucormycosis may affect the patients who underwent prolonged hospitalization and have been in touch with catheters and ventilators (Castrejón-Pérez et al., 2017).

The massive upsurge or according to local news “Tsunami of black fungus” has been observed in India in the wake of a spike in COVID-19 cases. Patients infected with this black fungus are presenting with nasal congestion, headache, and facial swelling (Serris et al., 2019). In the worst scenario, fever, cough, and dyspnea are also reported when the infection reaches the lungs (Serris et al., 2019). As per the reports, more than 10,000 patients have been known to be infected with black fungus (mucormycosis) in different parts of India (Bhuyan, 2021; Moona and Islam, 2021). Statisticians have claimed that steroids useful in curtailing the mortality rate in COVID-19 patients are potential factors for mucormycosis infection (Mehta and Pandey, 2020). At the same time, the shortage of oxygen tanks and delivery devices due to fulfilling the demands of exceedingly high COVID-19 cases in India has compelled the local authorities to gather oxygen cylinders without keeping an eye on their sources, resulting in the use of outdated oxygen delivery devices and cylinders contaminated with black fungus colonies delivered to the local hospital authorities (Carter and Notter, 2021; Feinmann, 2021). Other possible risk factors could be steam inhalation abuse, genetic pre-disposition, higher use of antibiotics, poor oral-nasal hygiene, repeated use of the same mask, etc. (Spellberg et al., 2012).

There are not much data available on the treatment and preventive measures for black fungus so far; however, experts are only left with either advising the use of Amphotericin B and posaconazole, or isavuconazole, or mostly the employment of surgical procedures to remove infected or dead tissue (Ochi et al., 1988).

## Impacts of COVID-19

COVID-19 has frequently affected day-to-day life and decelerated the global economy by freezing world trade and moments in multiple ways (Chakraborty and Maity, 2020). Despite these, the biggest threat that the world is facing today is to slow down the mortality rates due to the rapid spread of SARS-CoV-2 and its associated manifestations. The complications of modern medicine and research have been aggravated by the emerging variants of nCoV-2, influencing the responses of the drugs and vaccines designed so far (Munzig, 2019). There are reports available that evidence the long-term psychological impact of COVID-19 on people even after being cured of it (Saladino et al., 2020).

The havoc caused by the COVID-19 pandemic has pushed the world into prolonged exposure to stress due to a sense of helplessness, lack of freedom, and separation from our dear ones (Saladino et al., 2020). As an aftermath, psychological disturbances, depression, anxiety, and inability to tackle negative emotions leading to suicidal attempts have become a subject of concern (Saladino et al., 2020). The group which is majorly affected due to this is school-going children, college students, earning youngsters, and the health professionals (Saladino et al., 2020). As per a recent survey on 1143 parents and children aged between 3–18 years in Italy and Spain, parents experienced drastic emotional and behavioral changes in their children during lockdown (Orgilés et al., 2020). Parents reported that their children have difficulty in concentrating, few of them have expressed consistent irritability, complaining of boredom, sensing uneasiness, and loneliness throughout the quarantine (Orgilés et al., 2020). On contrary, the observations in the parents have exhibited some worrying responses, including: most of the parents have undergone depression due to loss of their wages and earning resources, in some cases relationships among the couples have also been compromised, and staying disconnected for longer (Orgilés et al., 2020). In corroboration to this, the data collected from a small survey in China during the initial stages of quarantine due to COVID-19 also demonstrated its worsening impact on the socio-behavioral and psychological tendencies of college-going students and earning individuals especially (Li et al., 2020a).

Apart from this, literature has also emphasized the mental state of healthcare workers (HCWs) and health professionals, who have equally been affected and registered with a high level of stress due to soaring COVID-19 cases (Garcia-Castrillo et al., 2020; Saladino et al., 2020). Ever since the emergence of SARS-CoV-2, this particular segment of people is engrossed with direct contact of the COVID-19 patients and loaded with enormous responsibilities, making them suffer from a high level of psycho-physical stress (Mohindra et al., 2020). This causes them to enter into secondary traumatic stress disorders, emotional and physical exhaustion, and sometimes a sense of helplessness is observed when sufficient resources and treatments lack to save lives (Mohindra et al., 2020; Saladino et al., 2020).

Therefore, to combat these alterations in the socio-psychological behavior of the majority of the population across the globe, World Health Organization (WHO) and Centers for Disease Control and Prevention (CDCP) have advocated specific conventions on the correct usage of health protection to curtail the distress and anxiety in the communities getting affected during the pandemic (Saladino et al., 2020). Besides this, local governments have also decided to avail psychotherapists to provide psychological support online and help the youngsters and HCWs to deal with their challenges (Saladino et al., 2020).

## Treatments Available

The global threat that prevailed due to this emerging SARS-CoV-2 virus has been a challenge to the health sector and medicine due to lack of information on specific anti-viral therapies or pharmacological entities to prevent nCoV-2 infection. As emergency medicine, doctors are relying on oxygen therapy, extracorporeal membrane oxygenation (ECMO), glucocorticoid supplementation (Dexamethasone), a common antibiotic, and antifungal treatments (van Paassen et al., 2020). Refer to **Table 1** (Han et al., 2020; Madjid et al., 2020; Zheng, 2020; Ardestani and Azizi, 2021; Malgotra and Sharma, 2021; Shamsi et al., 2021).

**Table 1:** The list of potential therapeutics against clinical manifestations of COVID-19 in use and their side effects.

S.No.	Drugs	Primary manufacturer (Year of approval)	Primary target condition	Route of administration	Mode of action	Recommended dose	Complications/Limitations
1.	Ribavirin (Avigan)	International Chemical and Nuclear Cooperation (ICN)/ Valeant pharmaceuticals (1970)	Pediatric respiratory syncytial virus (RSV) infection	Intravenous (I.V.)	Inhibits both RNA and DNA viruses, prevents mRNA capping, and has anti-MERS-CoV activity as well.	Adults: 500 mg/time, twice or thrice a day for not more than 10 days.	Anxiety, insomnia, possibility of hemolytic anemia, etc.
2.	Remdesivir (RDV)	Gilead Sciences, Inc. (2009)	Hepatitis C virus (HCV) and RSV	Intravenous (I.V.)	Halts viral genome replication by inhibiting RNA-dependent RNA polymerases (RdRp) activity.	Adults:100 mg once daily for 5–10 days.	Black fungus, altered blood pressure, nausea, etc.
3.	Lopinavir-Ritonavir	Lopinavir by Abott laboratories (1997) and Ritonavir by Abb Vie Inc. (1996)	Human immunodeficiency virus (HIV)	Oral	Inhibits cytochromes (CYP3A4 and CYP2D6) and P- glycoprotein. Prevents Gag-Pol polyprotein breakdown and thereby increases plasma concentrations of anti-viral drugs.	Adults: 400 mg/100 mg each time, for not more than 10 days.	Muscle cramps, vomiting, acidity, insomnia, etc.
4.	Chloroquine	Discoverer and primary manufacturer- Bayer laboratories (1934)	Malaria	Oral	Interferes with endocytic pathway, seizes sialic acid receptors, resists pH mediated spike (S) protein cleavage at hACE-2 binding site and prevents cytokine storm.	Adults: 500 mg/d, orally, 5–7 days, in addition to standard care.	Loss of appetite, weakness, skin rashes, itching, etc.
5.	Darunavir-Cobicistat	Darunavir- Tibotec pharma. In association with Johnson & Johnson (marketed in 2006) and Cobicistat- Janssen pharmaceuticals (2012)	Human immunodeficiency virus (HIV)	Oral	Selectively blocks the cleavage of viral-encoded Gag-Pol polyprotein in virally infected cells, thereby inhibiting viral replication	Adults: 400 mg and 100 mg, respectively, twice a day for 14 days, in addition to standard care.	High blood pressure, frequent urination, clay colored stool, possibility of jaundice, etc.
6.	Favipiravir	Glenmark pharmaceuticals (2014)	Human influenza A and B infections	Oral	Halts viral genome replication by inhibiting RNA-dependent RNA polymerases (RdRp) activity.	Adults: 1800 mg bid on day 1, followed by 800 mg bid on days 2–14.	Diarrhea, elevated uric acid, reduced leukocytes, etc.
7.	Abidol	USSR Research Center for Medical Chemistry (2010-2011)	Human influenza A and B infections	Oral	Prevents the fusion of viral lipid membranes with host cell surface receptors, and thereby inhibits replication.	Adults: 200 mg, twice for 5 d.	Constipation, nausea, dizziness, vomiting, etc.
8.	Oseltamivir (Tamiflu)	Gilead Sciences (2015)	H1N1 flu and its subtypes	Oral	Selectively inhibits neuraminidase enzyme and thereby preventing viral entry to host cells, viral release from infected cells, and further spread in the body.	Adults: 75 mg, twice for 10 d.	Insomnia, eye redness, nose bleeding, dizziness, and diarrhea mainly.
9.	ASC09F	Ascletis Pharmaceuticals Co., Ltd (under trials)	HIV protease inhibitor	Oral	Inhibits viral proteases and in turn blocks viral replication.	Adults: 400 mg, twice for 14 d.	Hepatotoxicity, retinal damage, nephrotoxicity, and cardiotoxicity.
10.	2-Deoxy-D-glucose	Institute of Nuclear Medicine & Allied Sciences (INMAS), Defence Research and Development Organization (DRDO)- Dr. Reddy’s Laboratory (2021)	Tumor	Oral	It accumulates selectively in the virus-infected cells and prevents viral replication by suppressing glycolysis by competitively inhibiting hexokinase 2 (HK2) as minimizing ATP synthesis for viral synthesis within the host cell.	Adults: 2.34 g, in dissolved form (water), for 10 d.	Still under trials, cannot be recommended to people with respiratory distressed conditions, allergic, or high diabetes.

The increasing number of cases and deaths due to COVID-19 has raised the concerns of local governments in all the nations across the globe. This has further created immense pressure on researchers and clinicians to expand trials on unknown or new drug moieties. According to the undergoing clinical trials the Food and Drug Administration (FDA) has recommended and allotted **“emergency use authorization (EAU)”** certification only to the three types of vaccines:

**1. mRNA-based vaccines:** exhibit an encouraging alternative to conventional vaccine approaches with their expeditious development capacities, low manufacturing investments, high potency, and secure administration (Verbeke et al., 2021). mRNA vaccines carry material from the COVID-19 causing virus that instructs our cells to synthesize a harmless protein that is unusual to the virus. Once the copies of such proteins are prepared by the cells, the genetic material from the vaccine is destroyed. Immediately after this body recognizes no more need for those proteins, so it starts generating relevant T- and B- lymphocytes to store the memory of how to combat the encounter of the body with the same virus for the next time (Verbeke et al., 2021).
**2. Subunit vaccines:** Protein subunit vaccines also called “A cellular vaccines” are considered to be the safest vaccine types over other categories. Instead of injecting a whole pathogen to trigger an immune response, they include purified, harmless protein fragments of the viral or bacterial pathogen selected especially for stimulating immune cells. After having sufficient copies of the desirable protein, our body cells then promotes the production of T- and B-lymphocytes for the memory to protect the body from future attack of the same virus (Kaur and Gupta, 2020).
**3. Viral vector-based vaccines:** Viral vector vaccines employ live viruses to convey DNA into host cells for synthesizing antigenic proteins that can further be tailored to trigger the range of immune responses, especially the production of antibodies, cytotoxic T lymphocytes, T-helper cells, etc. (Crommelin et al., 2021). In other words, such vaccines generally embody live attenuated viruses which can genetically be engineered to carry DNA encoding antigens (protein) from a different organism (Crommelin et al., 2021). Therefore, with the combined efforts of research and development (R&D) sectors at present, we have some efficient anti-viral vaccines from different pharmaceuticals to guard us against the pandemic. Refer to **Table 2** (Food and Drug Administration, 2019; Chagla, 2021; Kashte et al., 2021; Kumar et al., 2021).

**Table 2:** Update on FDA authorized COVID-19 vaccines for emergency use.

S.No.	Vaccines (Code names)	Manufacturer	Type of the vaccine	Efficacy rate	Doses/Shots	Side effects	FDA authorization/Approval
1.	BNT162b2	Pfizer- BioNTech Ltd., USA	mRNA-based vaccine	94–95%	2 shots (21 days apart)	Site of vaccination-pain, erythema, swelling. General side-effects: tiredness, muscle pain, headache, nausea, and mild fever for few days.	Yes (Emergency Use Authorization (EUA))
2.	mRNA-1273	Moderna TX, Inc., USA	mRNA-based vaccine	94–95%	2 shots (28 days apart)	At the place of vaccine: pain, redness, swelling of the lymph node. General side-effects: severe allergic reaction, fatigue, muscle pain, headache, vomiting, and mild fever for few days.	Yes (Emergency Use Authorization (EUA))
3.	JNJ-78436735	Johnson & Johnson’s- Janssen pharmaceuticals, USA	Viral vector-based vaccine	75%	1 shot	At the place of vaccine: pain, redness, swelling of the lymph node. General side-effects: fatigue, muscle pain, headache, vomiting, and mild fever for few days.	Yes (Emergency Use Authorization (EUA))
4.	AZD1222 (Covishield- India And Vaxzevria- Europe)	Oxford-AstraZeneca, USA and Serum Institute, India	Viral vector-based vaccine	64%	2 shots (28 days apart)	At the place of vaccine: pain, redness, swelling of the lymph node. General side-effects: vomiting, diarrhea, dizziness, etc. insomnia for few days to weeks.	Yes (Emergency Use Authorization (EUA))
5.	NVX-CoV2373	Novavax and Coalition for Epidemic Preparedness Innovation (CEPI)	Subunit and viral vector-based vaccine	89%	2 shots (28 days apart)	At the place of vaccine: pain, redness, swelling of the lymph node. General side-effects: vomiting, diarrhea, dizziness, etc. insomnia for few days to weeks.	Yes (Emergency Use Authorization (EUA))
6.	Sputnik V	Gamaleya Research Institute, Russia	Viral vector-based vaccine	92%	1 shot	Site of vaccination: pain, erythema, swelling. General side-effects: tiredness, muscle pain, headache, nausea, and mild fever for few days.	Yes (Emergency Use Authorization (EUA))
7.	BBV152/ Covaxin	Bharat Biotech, India	Inactivated vaccine	81%	2 shots (28 days apart)	At the place of vaccine: pain, redness, swelling of the lymph node, itching. General side-effects: mild fever, vomiting, diarrhea, malaise, dizziness, etc. insomnia for few days to weeks.	Pending

Almost a year after the start of the COVID-19 pandemic, the U.S. Food and Drug Administration authorized the emergency use of the first nCoV-2 vaccine (Quinn et al., 2020). Raising vaccines and conducting vaccination drives has been realized to be the foremost requisite of public health intervention. Indeed, it is the only way to stay protected during the pandemic. Again, this incredible and prolific outcome of researchers’ great input has been found to be associated with a limitation, known as “vaccine hesitancy” (Sallam, 2021). According to the World Health Organization (WHO), vaccine hesitancy is a retarded acceptance or rejection of vaccine despite the ongoing vaccination drives; it has been labeled as among the top-10 global threats of 2019 (Recio-Román et al., 2021). A significant proportion of the U.S. population has experienced a pattern of hesitancy against this first COVID-19 vaccine (Coustasse et al., 2021). This skeptical attitude of individuals against the non-authorized SARS-CoV-2 vaccine has been more aggravated by the previous experience from the approved flu A and B vaccine reported with minimal acceptance and in turn introduced an anonymous distrust among the community (Razai et al., 2021). Additionally the political influence and fake media announcements have also played a considerable role in manipulating the notions of the general public on available COVID-19 vaccine (Mills et al., 2020; Razai et al., 2021). As per a small survey conducted on 10-item vaccine hesitancy scale developed by WHO SAGE working group in Canada, China, Ethiopia, and Guatemala, maximum hesitancy towards any novel vaccine is expressed by parents of the middle age group (Wagner et al., 2021). Around 19% of the total parents within any country have expressed their distrust against un-authorized or non-FDA approved vaccines, making the vaccination drive challenging (Wagner et al., 2021).

Indeed the rationale behind vaccine hesitancy has been categorized into three categories: (1) lack of trust on the efficacy and safety profile of the vaccine, (2) missing complacency, i.e., confidence on not perceiving vaccine preventable diseases (VPDs) in the future, and (3) inconvenience due to managing authorities responsible for vaccination in terms of accessibility and unavailability of vaccine, unorganized vaccination pattern, inequitable distribution of vaccine, appeal of immunization services such as time, place, language, and cultural contexts (Razai et al., 2021).

Therefore, to overcome this COVID-19 induced catastrophic moral failure of the world, first we need to untidily work on pushing the equitable production and equitable distribution of vaccine to every single individual across the globe at our earliest convenience. Second, we need to keep on track on boosting our testing and tracing, oxygen supplies, and therapeutic and public health measures approaches.

## Concluding Remarks and Future Perspective

As the COVID-19 pandemic continues to unfurl, the complications of the general public and health sector crisis are presumed to continue. A subsequent increase in cases and flaring death cases due to the outbreak of this disease created unprecedented havoc initially in most afflicted nations such as China, Italy, the United States, and Iran, and eventually to the entire world. Earlier the WHO and local governments of all the countries decided to impose global lockdown to minimize social gatherings and in-person contact with the thought of preventing SARS-CoV-2 distribution. Later it was reported that the virus can spread even through the aerosols in the atmosphere and can be transmitted from pets to humans and vice versa. This has further compounded an inevitable stress on researchers and doctors and poses a serious threat to public health.

Henceforth, the need for enormous research and meta-analysis was realized and the significant findings from those studies to justify the reason for viral spread, possible preventive measures, and future approaches to be adopted made available to the general audience in the form of distinguished articles in pieces. Therefore, to save the precious time and effort of our readers, the authors in the present review have strived to gather, compile, and place every possible detail on COVID-19 disease from different sources and available literature in one place. By diving deep into the details shared in retrospective cohort studies and considering the present scenario, the current review has intensively discussed the origin of COVID-19, its pathogenesis, epidemiology, possible routes of SARS-CoV-2 invasion and zoonotic dissemination, virus variants, its implications on mental as well as on physical health, post-COVID-19 side-effects, and details on pros and cons of available curatives (therapeutics and vaccines) across the globe.

Depending on the rate of mortalities and the catastrophe caused by nCoV-2 infection, the production and administration of vaccines should be prioritized by limiting the usage of corticosteroids to COVID-19 patients to prevent the ferocious side effects, especially mucormycosis infection. We should also speed our trials on ongoing therapeutics with a special emphasis on some minimally studied nanoparticles, bio-flavonoids, nano-nutraceuticals, etc., with reported antiviral attributes to obtain certain novel and efficient pharmacological moieties to combat COVID-19 and post-COVID-19 clinical manifestations with minimal or no side effects.

## Author Contributions

RR contributed to the study design and concept. RR, AT, NK and NG were involved in the study organization. RR and AT wrote the manuscript. All authors contributed to the article and approved the submitted version.

## Conflict of Interest

The authors declare that the research was conducted in the absence of any commercial or financial relationships that could be construed as a potential conflict of interest.

## Publisher’s Note

All claims expressed in this article are solely those of the authors and do not necessarily represent those of their affiliated organizations, or those of the publisher, the editors and the reviewers. Any product that may be evaluated in this article, or claim that may be made by its manufacturer, is not guaranteed or endorsed by the publisher.

## Abbreviations

ACE, Angiotensin-converting enzyme; β-CoV, Betacoronavirus; BatCov, Bat coronavirus (HKU3); CoV, Coronavirus; COVID-19, Coronavirus disease 2019; FDA, Food and Drug Administration; GMT, Greenwich Mean Time; HCoV, Human coronavirus; hACE, Human Angiotensin-converting enzyme; HIV, Human Immunodeficiency Virus; ILs, Interleukins; IFN, Interferon; WHO, World Health Organization; MERS, Middle East respiratory syndrome; SARS, Severe Acute Respiratory Syndrome; TMPRSS2, Transmembrane protease, serine 2.
